# Quantitative Analysis of Isoniazid and Its Four Primary Metabolites in Plasma of Tuberculosis Patients Using LC-MS/MS

**DOI:** 10.3390/molecules27238607

**Published:** 2022-12-06

**Authors:** Nguyen Ky Anh, Pham My Tung, Min Jung Kim, Nguyen Phuoc Long, Yong-Soon Cho, Dong-Hyun Kim, Jae-Gook Shin

**Affiliations:** 1 Department of Pharmacology and PharmacoGenomics Research Center, Inje University College of Medicine, Busan 47392, Republic of Korea; 2 Center for Personalized Precision Medicine of Tuberculosis, Inje University College of Medicine, Busan 47392, Republic of Korea; 3 Department of Clinical Pharmacology, Inje University Busan Paik Hospital, Busan 47392, Republic of Korea

**Keywords:** tuberculosis, metabolic profiles, isoniazid, hydrazine, *NAT2*, mass spectrometry

## Abstract

Isoniazid and its metabolites are potentially associated with hepatotoxicity and treatment outcomes in patients who receive antituberculosis (TB) therapy. To further understand the pharmacokinetic profiles of these molecules, a method based on LC-MS/MS was developed to determine the concentration of these compounds in human plasma. Isoniazid, acetylisoniazid, and isonicotinic acid were directly analyzed, whereas hydrazine and acetylhydrazine were determined after derivatization using p-tolualdehyde. Chromatographic separation was conducted on reversed-phase C18 columns with gradient elution, and detection was carried out in multiple reaction monitoring mode. The calibration curves were linear with correlation coefficients (r) greater than 0.9947 for all analytes. The intra- and inter-day precision was less than 13.43%, and the accuracy ranged between 91.63 and 114.00%. The recovery and matrix effect of the analytes were also consistent (coefficient of variation was less than 9.36%). The developed method successfully quantified isoniazid and its metabolites in TB patients. The method has broad applications in clinical research, including isoniazid one-point-based therapeutic drug monitoring, genotype–phenotype association studies of isoniazid metabolic profile and isoniazid-induced hepatotoxicity, and the initial dose prediction of isoniazid using population pharmacokinetic modeling.

## 1. Introduction

Tuberculosis (TB), a global health issue, was responsible for approximately 1.2 million deaths in 2021, as estimated by the World Health Organization [1]. Isoniazid (INH) is a first-line agent in part of a multidrug therapy often used for the treatment of TB. Although INH is a highly effective drug, INH treatment is also associated with abnormal elevation of alanine aminotransferase (ALT) levels in approximately 20% of patients and hepatotoxicity in up to 1% of patients taking the drug [2,3,4]. According to studies in animal models, INH-induced hepatotoxicity is thought to be related to hydrazine (HZ) and acetylhydrazine (AcHZ), two reactive metabolites [5,6]. However, other lines of evidence indicated that INH itself could also be the cause of hepatotoxicity [7,8,9]. The pharmacokinetic profiles of INH and its metabolites vary considerably between ethnic populations, pharmacogenetics, and comorbidities [10,11,12,13]. Furthermore, they are also related to therapeutic success [14]. Therefore, the characterization of INH and its metabolite profile will provide more insights into interindividual differences in INH exposure, thus contributing to the implementation of precision dosing.

**Figure 1:**
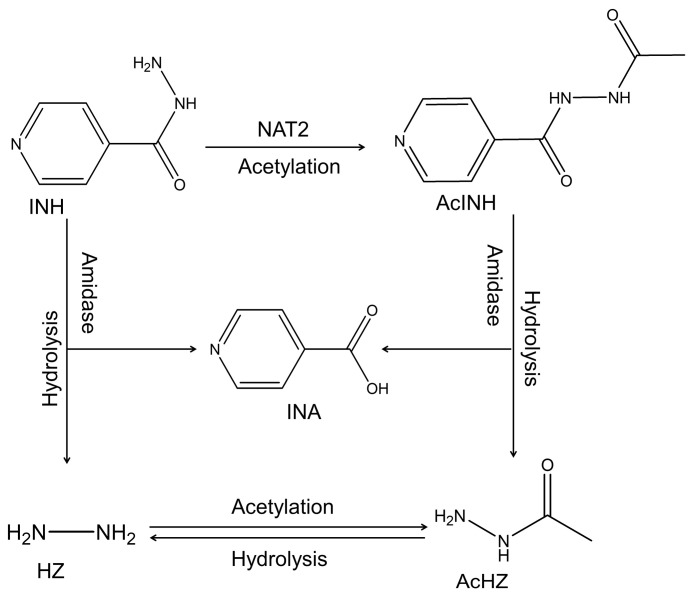
Chemical structures and metabolic pathway of INH and metabolites. Compounds that are quantified in this study are INH, AcINH, INA, HZ, and AcHZ. Abbreviations: INH, isoniazid; AcINH, acetylisoniazid; INA, isonicotinic acid; HZ, hydrazine; AcHZ, acetylhydrazine; NAT2, *N*-acetyltransferase-2.

In the liver and intestines, INH is mainly metabolized by *N*-acetyltransferase-2 (*NAT2*), which acetylates it to form *N*-acetylisoniazid (AcINH) [15]. Both INH and AcINH can be hydrolyzed into HZ and AcHZ, respectively, by amidase. This hydrolysis reaction also yields isonicotinic acid (INA). AcHZ can be deacetylated to HZ via hydrolysis by amidase or further acetylated by *NAT2* to diacetylhydrazine [15,16]. The chemical structures and the metabolic pathway of INH and its metabolites are presented in Figure 1.

Several methods have been developed to quantify INH and metabolites in human plasma using liquid chromatography–tandem mass spectrometry (LC-MS/MS) [17,18,19,20,21,22]. These studies used a variety of methodologies for sample preparation and chromatographic separation. In the case of sample preparation, solid-phase extraction [17], liquid–liquid extraction [18], or single-step protein precipitation [19,22] were used. For the chromatographic separation, various methodologies were applied, from reverse phase C18 [17,19,20,23] to hydrophilic interaction liquid chromatography (HILIC) [18]. Regarding the calibration range, although previous studies well cover the low concentration range of the analytes, the upper limit of quantification may not reach the clinically high concentration range of INH and metabolites in some cases [17,20]. This may lead to routine sample dilution, which requires more sample volumes, more time, and more cost.

Unlike INH, AcINH, and INA, it is not feasible to directly quantify HZ and AcHZ using LC-MS/MS in human plasma [20]. Due to low molecular weight, it is unable to obtain stable product ions of AcHZ and HZ in tandem MS while only detecting the precursor ions is not sufficient to confirm the analyte in a complex matrix. Therefore, different strategies should be applied to measure INH and metabolites using LC-MS/MS, a direct approach for INH, AcINH, and INA, and a derivatization approach for HZ and AcHZ. In this study, our aim was to develop a simple and accurate quantitative method to determine the plasma concentration of INH and its four metabolites using LC-MS. The method was validated for selectivity, linearity, accuracy, precision, and matrix effect according to the FDA guidance for bioanalytical method validation [24]. Our method was applied to analyze plasma samples of TB patients who took INH for disease management for one-point-based therapeutic drug monitoring (TDM). Plasma concentrations of the analytes were also compared with *NAT2* functional phenotypes.

## 2. Results and Discussion

### 2.1. Method Optimization for LC and MS Conditions

In this study, we used the multiple reaction monitoring (MRM) mode to quantify INH and four metabolites in human plasma. Analytes were divided into two groups. INH, AcINH, and INA (group I) were analyzed directly after protein precipitation. HZ and AcHZ (group II) were analyzed after derivatization with p-tolualdehyde to improve their separation and detection in the LC-MS/MS system. All analytes generated the major protonated molecular ions [M + H]^+^ in positive ion mode, and those ions were selected as the precursor ions. For the transition in the subsequent MRM mode of group I, the most abundant fragment ions in the MS/MS spectra of the analytes were chosen as the product ion. These transitions are consistent with previous reports [17,18,19]. For group II, the transitions for MRM mode followed the setup of Lu Song et al. [20]. The MRM transitions and optimized collision-induced dissociation conditions are given in Table 1.

Various sample preparation methods have been reported for LC-MS/MS analysis of INH and its metabolites, such as solid-phase extraction, liquid–liquid extraction, and protein precipitation [17,18,19]. Protein precipitation using an organic solvent is simple, robust, and cost-efficient. Therefore, we utilized single-step protein precipitation using ACN for group I and MeOH for group II. For chromatographic separation, two C18 columns were selected based on their previous performance in quantifying INH and its hydrazine metabolites [20,25]. The internal standards were included to control for shifts in retention time, as previously shown to be suitable for INH quantification using LC-MS/MS [26].

### 2.2. Method Validations

#### 2.2.1. Selectivity, Linearity, and Carryover

**Figure 2:**
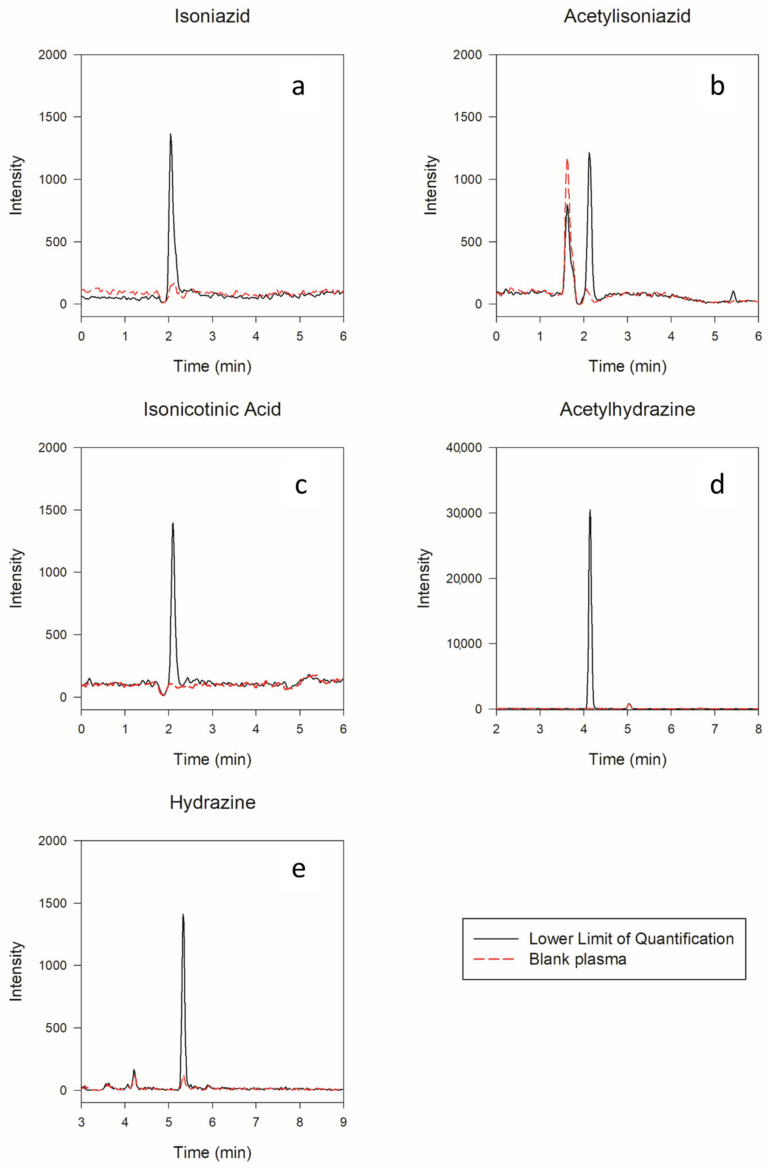
Chromatogram of all analytes in LLOQ compared to blank plasma. There were no significant interferences at the time of the analyte’s elusion in blank plasma, indicating the method is specific for each analyte: (**a**) isoniazid LLOQ compared to blank plasma; (**b**) acetylisoniazid LLOQ compared to blank plasma; (**c**) isonicotinic acid LLOQ compared to blank plasma; (**d**) acetylhydrazine LLOQ compared to blank plasma; (**e**) hydrazine LLOQ compared to blank plasma.

The method’s selectivity was evaluated by preparing and analyzing human plasma blanks from six individual batches and comparing them with spiked plasma samples. No interferences were detected at the elution times of the analytes and IS. The chromatogram of blank plasma and LLOQ samples is shown in Figure 2.

The calibration ranges for each analyte were selected as suitable for monitoring plasma analyte concentrations based on their reported pharmacokinetic profiles (Table 1) [10,27,28]. All analyte calibration curves were processed by a weighted least-squares linear regression model. Two weighting factors, 1/x^2^ and 1/x, were the best fit for groups I and II, respectively. The calibration curves for all analytes were linear over the concentration ranges, with correlation coefficients (r) ≥ 0.9947 (Table 1). Signal-to-noise ratios at LLOQ were ≥10 for all analytes. No significant carryover effects were observed.

#### 2.2.2. Accuracy and Precision

The method shows acceptable intra- and inter-day variations (Table 2). The intra-day coefficients of variation were between 1.12 and 13.43%, and accuracies ranged from 91.63 to 114.00%. The inter-day coefficients of variation were between 2.49 and 9.69%, and the accuracy was between 97.46 and 108.78%.

#### 2.2.3. Matrix Effects and Recovery

The matrix effects and recovery of each analyte were evaluated at low and high QC concentrations (Table 3). Ion suppression was observed in all group I’s compounds (36.42–54.08%) but was consistent between different matrix sources (RSD ≤ 7.50%). The suppression could be explained by the coelution of these analytes with endogenous compounds in reversed-phase chromatography. Although there are substantial matrix effects, good accuracy and the precision of group I’s analytes indicated that it might not affect the quantification of those compounds. Moreover, the recovery ranged from 66.94 to 112.19% and was consistent with RSD ≤ 9.36%.

#### 2.2.4. Stability

The stability of five analytes in various conditions is summarized in Table 4. All compounds are stable in human plasma at room temperature up to 4 h (accuracy from 86.65 to 113.67%). The post-extraction samples of all compounds in group I and the derivatization products of group II were also stable in the autosampler at 4 °C for 24 h (accuracy from 92.86 to 104.46%) and 18 h (accuracy from 83.83 to 111.33%), respectively. After three freeze–thaw cycles, the compounds showed no significant degradation except for INH and INA in low concentrations [19]. The stability results indicated that human plasma samples should be prepared within 4 h under room temperature conditions and could be stored at 4 °C up to 18 h. Freeze–thaw cycles should be limited to avoid compound degradation.

### 2.3. Application to Clinical Research

Although we applied two strategies for a comprehensive assessment of INH metabolites in plasma from patients with TB, each strategy is fully validated and could be used as a separate method depending on the purpose and availability. For example, the direct quantification strategy of INH, AcINH, and INA could be used for TDM or a potential alternative way of phenotyping *NAT2* function in various settings due to its simplicity. In addition, the quantification strategy for HZ and AcHZ could be used alone for toxicity research into INH or in combination with the other strategy for studying INH metabolism. In this study, the developed method was successfully applied to measure INH and its metabolite concentrations in 96 TB patients who received four first-line anti-TB drugs in the cPMTb cohort.

**Figure 3:**
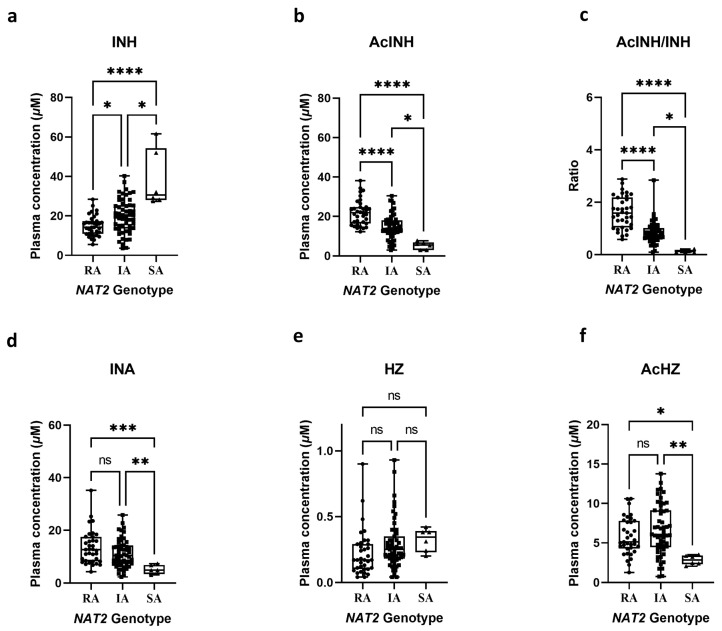
Concentrations, ratio of isoniazid and four metabolites in tuberculosis (TB) patients’ plasma at 2 h after drug administration according to N-acetyltransferase-2 (*NAT2*) phenotypes: (**a**) isoniazid 2 h postdose plasma concentration in TB patients according to *NAT2* phenotypes; (**b**) acetylisoniazid 2 h postdose plasma concentration in TB patients according to *NAT2* phenotypes; (**c**) acetylisoniazid/isoniazid 2 h postdose plasma ratio in TB patients according to *NAT2* phenotypes; (**d**) isonicotinic acid 2 h postdose plasma concentration in TB patients according to *NAT2* phenotypes; (**e**) hydrazine 2 h postdose plasma concentration in TB patients according to *NAT2* phenotypes; (**f**) acetylhydrazine 2 h postdose plasma concentration in TB patients according to *NAT2* phenotypes. Abbreviations: INH, isoniazid; AcINH, acetylisoniazid; INA, isonicotinic acid; HZ, hydrazine; AcHZ, acetylhydrazine; *NAT2*, *N*-acetyltransferase-2; RA, rapid acetylator; IA, intermediate acetylator; SA, slow acetylator; ns, *p* > 0.05; *, *p* ≤ 0.05; **, *p* ≤ 0.01; ***, *p* ≤ 0.001, ****, *p* ≤ 0.0001.

As previously reported, the pharmacokinetics profiles of INH and its metabolites exhibit wide interindividual variability among *NAT2* functional phenotype groups [11,29]. In general, rapid acetylators are prone to unfavorable outcomes due to subtherapeutic exposure to the parent drug [30,31,32]. On the contrary, slow acetylators may develop hepatotoxicity as a consequence of greater exposure to INH toxic metabolites [7,33]. In fact, clinical studies have discussed the benefit of *NAT2*-genotype-guided precision dosing to balance between the effectiveness and safety of INH-based therapy [34,35]. Understanding the correlation between INH metabolism and *NAT2* functional phenotypes in different ethnic would facilitate the implementation of genotype-based precision medicine for INH-based therapy. Therefore, the INH metabolic profiles of patients at 2 h postdose were compared with *NAT2* functional phenotypes. The patients were classified into three *NAT2* acetylation phenotypes: rapid acetylator (*n* = 35), intermediate acetylator (*n* = 55), and slow acetylator (*n* = 6), according to their respective genotypes [36]. Overall, the analyte concentrations at 2 h postdose were within the calibration range of the method. Furthermore, the agreement in INH metabolite concentrations and the *NAT2* phenotype of TB patients determined by the SNaPshot multiplex genotyping assay further emphasizes the accuracy of the developed assay (Figure 3). Briefly, patients with the *NAT2* slow acetylator have a significantly lower concentration of acetylated metabolites such as AcINH and AcHz than in other groups, while the parent drug concentration is significantly higher. The concentration of HZ, the metabolites related to INH hydrolysis, showed the most negligible dependence on *NAT2* phenotypes since amidase is responsible for its formation [15]. In fact, the interindividual variability in INH and its metabolites at 2 h postdose could be explained by the difference in the *NAT2* enzyme function level between subjects, as reported by previous findings [29,37].

## 3. Materials and Methods

### 3.1. Chemicals and Reagents

INH, p-tolualdehyde, phenacetin, sulfamethazine, and formic acid were obtained from Sigma-Aldrich (St. Louis, MO, USA). AcINH, AcHZ, INA, and HZ hydrochloride were purchased from Toronto Research Chemicals (North York, ON, Canada). HPLC-grade acetonitrile (ACN) and methanol (MeOH) were obtained from Avantor (Radnor, PA, USA). Water was purified by Milli-Q Plus water purification system (Merck, Darmstadt, Germany). Drug-free human heparinized plasma was provided by Busan Paik Hospital (Busan, Republic of Korea). All other chemicals were of the highest analytical grade available.

### 3.2. Preparation of Standards and Quality Controls Samples

Stock solutions of INH, AcINH, and internal standards (phenacetin, sulfamethazine) were prepared in ACN, while the stock solution of INA, HZ, and AcHZ were prepared in MeOH. The stock solutions were stored at −20 °C prior to analysis. These stock solutions were then divided into two groups according to their quantification strategy; group I included INH, AcINH, and INA, and group II included HZ and AcHZ. Two working solutions were prepared by mixing the stock solutions of each group, followed by serial dilution to the required concentrations with ACN and MeOH for group I and group II, respectively. Calibration standard samples were prepared by spiking an appropriate amount of analyte to blank plasma for a total of seven concentrations. Quality control (QC) samples were freshly prepared for each batch in low, mid, and high concentrations.

### 3.3. Sample Preparation

Different extraction methods were applied for groups I and II. For group I, the analyte was extracted from plasma using simple protein precipitation. A total of 100 µL ACN containing 200 ng/mL phenacetin as internal standards was added to 50 µL plasma as a precipitation agent. The mixture was vortexed for 5 min and centrifuged at 9000× *g*, 4 °C for 10 min. Then, 50 µL of supernatant was taken and further diluted with 150 µL water containing 0.1% FA, after which 2 µL of aliquot was injected into the LC-MS/MS system.

For group II, sample preparation and derivatization were based on the method of Lu Song et al. with modifications [20]. Briefly, 100 µL of MeOH containing 50 ng/mL sulfamethazine as the internal standard was added to 50 µL of plasma for protein precipitation. Then, the samples were vortexed for 5 min. After centrifugation at 9000× *g* for 10 min at 4 °C, 100 µL of the supernatant was taken, and 10 µL of p-tolualdehyde was added with a concentration of 11.5 mg/mL in MeOH. The derivatization reaction was performed in ultrasonication (40 kHz) for 50 min at room temperature. Afterward, the samples were evaporated using a gentle nitrogen flow at room temperature and then redissolved in 100 µL of distilled water containing 5% ACN. After vortexing, the samples were transferred to a vial for LC-MS/MS analysis.

### 3.4. Liquid Chromatography–Tandem Mass Spectrometry

LC-MS/MS included an Agilent 1200 series HPLC system (Agilent, Wilmington, DE, USA) equipped with an autosampler, binary pump, and column oven, which was coupled with an API 4000 triple-quadrupole mass spectrometer (AB SCIEX, Framingham, MA, USA) equipped with a Turbo Ion Spray source. The Electrospray Ionization was set in positive ion mode: the voltage value was 4800 V, gas 1 and gas 2 were set at 40 psi, and the curtain gas at 30 psi. All compounds were quantified in multiple reaction monitoring (MRM) mode. The mass transition of each analyte and the optimal mass spectrometry parameters are described in Table 1. The mobile phases consisted of 0.1% formic acid in distilled water (A) and ACN containing 0.1% formic acid (B) at a flow rate of 0.2 mL/min for both groups. The samples of both groups I and II were stored at 4 °C in the autosampler during analysis time. Chromatographic methods of group I and group II were performed using reversed-phase C18 columns with gradient elution. In particular, an Atlantis dC18 column (150 × 2.1 mm, 3 µm; Waters, Milford, MA, USA) was used for separating compounds in group I. The autosampler and column oven were set at 4 °C and 35 °C, respectively. Gradient elution condition was set as follows: t = 0 min, A = 80%, B = 20%; t = 2 min, A = 20%, B = 80%; t = 5 min, A = 20%, B = 80%; t = 5.5 min, A = 80%, B = 20%; t = 12 min, A = 80%, B = 20%. For group II, a Waters X Bridge BEH C18 Column (100 mm × 2.1 mm, 2.5 μm; Waters, Milford, MA, USA) was used. The autosampler and column oven temperatures were maintained at 4 °C and 60 °C, respectively, during analysis. Elution conditions for group II was set as follows: t = 0 min, A = 95%, B = 5%; t = 1.5 min, A = 2%, B = 98%; t = 6 min, A = 2%, B = 98%; t = 6.5 min, A = 95%, B = 5%; t = 12 min, A = 95%, B = 5%.

### 3.5. Method Validation

The method validation was performed according to US Food and Drug Administration (FDA) guidance to validate bioanalytical methods [24]. The assessed parameters included selectivity, linearity, accuracy, precision, matrix effects, recovery, and stability in human plasma.

#### 3.5.1. Selectivity and Carryover

Selectivity was assessed by comparing six blank plasma samples from different individuals to samples spiked with analyte at the lower limit of quantification (LLOQ). The method is considered selective if there is no interference at the retention time of the target compounds. To assess possible carryover effects, a blank plasma sample was injected immediately after the highest calibration standard sample. Analyte’s response in blank plasma should not exceed ± 20% of LLOQ.

#### 3.5.2. Linearity and Lower Limit of Quantification

Calibration curves were obtained by analyzing standard plasma samples at seven analyte concentrations and processed by weighted least-squares linear regression. The weighting factor was tested between 1/x and 1/x^2^. The linearity of the calibration curve was determined by the correlation coefficient of the linear regression. The lower limit of quantification (LLOQ) for analytes in human plasma samples was defined as the lowest concentration detectable with a signal-to-noise ratio of at least 10, accuracy of 80–120%, and precision within 20%.

#### 3.5.3. Accuracy and Precision

For accuracy and precision, four levels of concentration were selected. Those levels were defined as LLOQ, low, medium, and high QC and were determined from the calibration range. A batch consisting of five replicates for each concentration was prepared to validate intra-day accuracy and precision. For inter-day validation, three freshly prepared equivalent batches were used for three separate days. Accuracy was calculated based on the relative error, and precision was calculated based on the relative standard deviation (RSD). The results were regarded as acceptable if they were in the range of ±15%, except for LLOQ, which is ±20% under FDA guidance.

#### 3.5.4. Matrix Effect and Recovery

Matrix effects were assessed in low, mid, and high QCs for group I by determining the peak area of post-extraction spiked supernatant (as A) and neat standard solution (water with 0.1% formic acid) (as B) containing equivalent concentrations of the analytes in six replications. The matrix effect was calculated by the following equation: Matrix effect (%) = (B – A)/B × 100.

The extraction recovery was evaluated by determining the peak area ratios between QC samples in low and high levels (pre-extraction spiked samples) and post-extraction spiked supernatant containing equivalent concentrations of analytes in six replications. The recovery was considered valid as long as it was consistent (RSD < 15%).

#### 3.5.5. Stability

The stability of samples on bench-top, post-extraction, and three freeze–thaw cycles were assessed at low and high concentration QCs in triplicates. Room temperature stability was assessed by keeping QC samples on the bench-top for 4 h before extraction. Post-extraction stability was evaluated by reanalyzing QC samples after 24 h storage at 4 °C. Three cycles of freezing at −80 °C for 24 h and thawing at room temperature were used to evaluate freeze–thaw stability. Adequate stability was defined as <15% loss of the initial analyte concentrations.

### 3.6. Clinical Application

The developed method was applied to determine the plasma concentrations of INH and its metabolites in 96 patients from a multicenter prospective cohort study in the Republic of Korea. The Center for Precision Medicine for Tuberculosis (cPMTb) cohort was designed to research and develop personalized pharmacotherapy implementations for TB [13,38]. Subjects were eligible if they met the following criteria: ≥21 years of age, diagnosed with TB, and currently under treatment with anti-TB drugs. The study was conducted following the Declaration of Helsinki and institutional criteria. Written informed consent was obtained from all subjects prior to obtaining blood samples.

### 3.7. Determination of NAT2 Genotype

Genomic DNA was extracted from whole blood using a Blood Genomic DNA Miniprep Kit (Cosmogenetech, Seoul, Republic of Korea) according to the manufacturer’s protocol. A single-base extension assay was conducted using SNaPshot Multiplex Kit (Applied Biosystems, Foster City, CA, USA) to analyze the six most common single-nucleotide polymorphisms (SNPs) sites (c.191G>A, c.282C>T, c.341T>C, c.590G>A, c.803A>G, and c.857G>A) in *NAT2* gene. The classification of *NAT2* acetylator phenotypes was performed using NAT2PRED [36].

### 3.8. Statistical Analysis

Statistical significance between two or three groups of acetylation status was determined by Kruskal–Wallis’s test using GraphPad’s Prism version 9.4.1. (Dotmatics, Boston, MA, USA). Differences were considered statistically different when *p*-value < 0.05.

## 4. Conclusions

We developed and validated an accurate yet straightforward quantification method utilizing LC-MS/MS to characterize the metabolic profile of INH in human plasma. According to the FDA guidance for the bioanalysis method, the developed method demonstrated satisfactory results in sensitivity, accuracy, and precision. The method showed technical feasibility, and the predefined analysis range also covers the concentration of each analyte in typical clinical scenarios. We successfully applied the method to measure plasma concentrations of INH and its metabolites in TB patients. The method could potentially facilitate one-point-based TDM, studies of the association between INH metabolic profile with genetic or INH-induced liver injury, and model-informed precision dosing of INH.

## Figures and Tables

**Table 1:** Multiple reaction monitoring parameters, calibration range, correlation coefficient, and IS for all five compounds.

Analytes	Transition (m/z)	DP (V)	CE (eV)	Calibration Range (ng/mL)	Correlation Coefficient	Internal Standard
INH	138 → 121	50	30	80–10,000	0.9970	Phenacetin
AcINH	180 → 121	60	30	80–10,000	0.9958	Phenacetin
INA	124 → 80	40	25	80–10,000	0.9947	Phenacetin
HZ	237.2 → 120	80	25	1–30	0.9975	Sulfamethazine
AcHZ	177 → 118	50	19	40–1200	0.9993	Sulfamethazine

Abbreviations: DP, declustering potential; CE, collision energy; V, volt; eV, electron volt; INH, isoniazid; AcINH, acetylisoniazid; INA, isonicotinic acid; HZ, hydrazine; AcHZ, acetylhydrazine.

**Table 2:** Intra- and inter-day accuracy and precision of all analytes.

Analytes	Nominal Conc. (ng/mL)	Intra-Day (*n* = 5)		Inter-Day (*n* = 3) *	
		RSD (%)	Accuracy (%)	RSD (%)	Accuracy (%)
INH	80	4.41	104.43	4.78	104.34
	240	3.92	107.78	5.29	103.84
	2000	5.21	106.16	3.01	103.10
	8000	7.93	105.84	3.30	101.69
AcINH	80	3.34	92.85	7.10	97.46
	240	11.57	95.38	4.67	98.74
	2000	3.73	99.10	4.93	99.25
	8000	4.59	97.20	5.43	99.89
INA	80	13.43	102.07	8.51	108.78
	240	10.54	91.63	9.69	103.01
	2000	9.31	96.00	2.87	97.89
	8000	8.74	100.94	8.95	100.59
HZ	1	5.58	112.00	9.36	106.26
	3	1.86	109.00	3.73	107.74
	12	6.28	98.10	6.49	100.36
	24	6.90	100.32	2.49	102.13
AcHZ	40	6.54	106.00	9.19	99.19
	120	1.12	114.00	8.22	106.07
	480	11.30	97.68	5.32	100.26
	960	9.00	100.30	5.60	100.67

Abbreviations: RSD, relative standard deviation; INH, isoniazid; AcINH, acetylisoniazid; INA, isonicotinic acid; HZ, hydrazine; AcHZ, acetylhydrazine. * *n* represents the number of days the test was conducted (3 d).

**Table 3:** Recovery and matrix effects of all analytes.

Analytes	Nominal Conc. (ng/mL)	Recovery (*n* = 6)		Matrix Effect (*n* = 6)	
		Mean (%) ± SD	RSD (%)	Mean (%) ± SD	RSD (%)
INH	240	92.38 ± 3.56	3.86	36.42 ± 2.55	7.01
	8000	96.92 ± 1.97	2.03	43.46 ± 3.08	7.09
AcINH	240	110.76 ± 3.05	2.75	53.88 ± 1.70	3.15
	8000	96.65 ± 1.23	1.27	54.08 ± 2.95	5.45
INA	240	66.94 ± 6.27	9.36	40.65 ± 1.67	4.12
	8000	78.17 ± 0.84	1.07	39.68 ± 2.97	7.50
HZ	1.5	109.67 ± 7.65	6.98	NA	NA
	24	70.35 ± 1.36	1.94	NA	NA
AcHZ	120	105.12 ± 2.42	2.30	NA	NA
	960	112.19 ± 1.64	1.46	NA	NA

Abbreviations: RSD, relative standard deviation; INH, isoniazid; AcINH, acetylisoniazid; INA, isonicotinic acid; HZ, hydrazine; AcHZ, acetylhydrazine; NA, not applicable.

**Table 4:** Stability of all analytes.

Analytes	Nominal Conc. (ng/mL)	Stability (%)		
		Bench-Top at RT (4 h)	Autosampler at 4 °C (*)	Freeze–Thaw (3 Cycles)
INH	240	103.82	104.46	77.36
	8000	93.84	93.55	92.83
AcINH	240	94.10	97.44	97.97
	8000	95.76	98.33	106.54
INA	240	92.93	92.98	79.44
	8000	108.23	92.86	113.96
HZ	3	86.65	91.07	87.13
	24	97.77	83.83	86.83
AcHZ	120	113.67	111.33	112.33
	960	112.00	101.00	107.00

Abbreviations: RSD, relative standard deviation; INH, isoniazid; AcINH, acetylisoniazid; INA, isonicotinic acid; HZ, hydrazine; AcHZ, acetylhydrazine. * 24 h for INH, AcINH, and INA or 18 h for HZ and AcHZ.

## Data Availability

The data of this study will be available upon reasonable request from the corresponding author. The data are not publicly available to protect the privacy of the participants.

## Sample Availability

Samples of the compounds (INH, AcINH, INA, HZ, and AcHZ) are available from the authors.
